# Lyme Disease Emergence after Invasion of the Blacklegged Tick, *Ixodes scapularis*, Ontario, Canada, 2010–2016

**DOI:** 10.3201/eid2502.180771

**Published:** 2019-02

**Authors:** Manisha A. Kulkarni, Isha Narula, Andreea M. Slatculescu, Curtis Russell

**Affiliations:** University of Ottawa, Ottawa, Ontario, Canada (M.A. Kulkarni, I. Narula, A.M. Slatculescu);; Public Health Ontario, Toronto, Ontario (C. Russell)

**Keywords:** Lyme disease, Borrelia burgdorferi, Ixodes scapularis, Canada, epidemiology, vector-borne disease, tickborne diseases, bacteria, blacklegged tick

## Abstract

Analysis of surveillance data for 2010–2016 in eastern Ontario, Canada, demonstrates the rapid northward spread of *Ixodes scapularis* ticks and *Borrelia burgdorferi*, followed by increasing human Lyme disease incidence. Most spread occurred during 2011–2013. Continued monitoring is essential to identify emerging risk areas in this region.

Lyme disease (LD) is the most reported vectorborne disease in North America, where it is caused by *Borrelia burgdorferi* sensu stricto and principally transmitted by the blacklegged tick (*Ixodes scapularis*) (*1*). With northward expansion of *I. scapularis* tick populations from endemic areas in the United States, LD is rapidly emerging in parts of central and eastern Canada (*2*–*4*). Although several studies have mapped blacklegged tick populations across Canada and developed models to predict future spread of ticks and LD risk (*2*,*3*), little is known about the extent of human LD in relation to tick vector distributions at a fine geographic scale. We examined spatiotemporal trends in the occurrence and expansion of *I. scapularis* ticks, *B. burgdorferi*–infected ticks, and human LD cases over a 7-year period to elucidate the process of LD emergence in eastern Ontario, Canada.

## The Study

Our study included 3 public health units in eastern Ontario, Canada: Kingston, Frontenac, and Lennox and Addington (KFL); Leeds, Grenville, and Lanark (LGL); and Ottawa. This region spans from the St. Lawrence River in the south to the Ottawa River in the north, and has several major population centers, including Kingston (2016 population 123,798) and Ottawa (2016 population 934,243) (*5*). The region is largely characterized by mixed deciduous forest and agricultural land use.

We used data from the Integrated Public Health Information System database to identify human LD cases on the basis of provincial case definitions (*6*). We geocoded cases to their forward sortation area (FSA) (i.e., first 3 digits of the postal code) of residence and extracted data on patient sex, age, episode date (onset of symptoms), and reported history of travel (defined as travel outside the municipality of residence within the previous 2 weeks). Data on ticks collected during 2010–2016 through passive tick surveillance activities in Ontario were obtained from Public Health Ontario (PHO) (*7*). We aggregated *I. scapularis* tick records according to the FSA of the submitter (i.e., location of residence of the person who acquired the tick) and excluded records with missing collection date, submitter FSA, or PCR test result and records with reported history of travel. We similarly excluded human LD records with missing patient FSA or with reported travel history. We obtained FSA-level population data for 2011 and FSA boundary files from Statistics Canada (*5*).

To examine the association between the invasion of *I. scapularis* ticks and *B. burgdorferi* and the spread of human LD, we examined associations between FSA-level data on time to first case (in years) and several variables: time to first reported *I. scapularis* tick, time to first reported *B. burgdorferi*–infected tick, distance to FSA with highest LD incidence in 2010, and population. We constructed bivariable and multivariable linear regression models with time to first case (in years) as the outcome.

To visualize LD spread during 2010–2016, we plotted the annual FSA-level incidence of human LD and *B. burgdorferi* prevalence in ticks by using ArcGIS 10.4 (ESRI, https://www.esri.com). We also assessed the annual weighted mean center and distribution of human LD incidence by using ArcGIS 10.4, after spatial projection of the data to preserve distance (*8*). We applied Kulldorff’s spatial scan statistics (*9*) by using SaTScan 9.6 (https://www.satscan.org) to assess and compare spatiotemporal patterns in human LD incidence and *B. burgdorferi* prevalence in ticks at the FSA level (FSA centroids). (For additional methods, see the Appendix).

**Figure 1:**
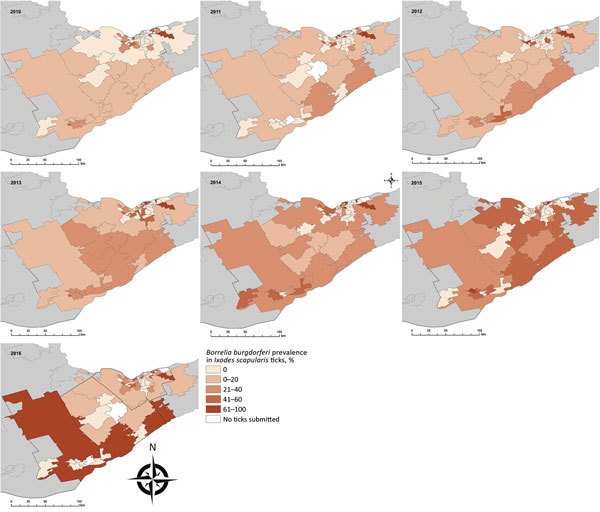
Annual prevalence of *Borrelia burgdorferi* in *Ixodes scapularis* ticks from passive tick surveillance, based on forward sortation area of tick submitter, 3 public health units, eastern Ontario, Canada, 2010–2016.

A higher proportion of LD cases occurred in men and in adults 50–69 years of age (Table 1), similar to patterns observed in other regions of North America (*10*). LD incidence increased over time; 55% of cases occurred during 2015 and 2016 (Table 2). Roughly 70% of cases occurred during June–August, whereas ≈20% occurred during September–December. The number of collected ticks increased annually from 2010 and reached a peak in 2013, with a subsequent decrease because of reductions in passive surveillance activities in KFL and LGL (*11*); Ottawa received an increasing amount of ticks over time (Table 2). The percentage of ticks testing positive for *B. burgdorferi* increased annually, from 12% in 2010 to 23% in 2016 (p<0.001). Infection rates were higher among regions of KFL and LGL, although FSAs with high *B. burgdorferi* prevalence among submitted ticks were observed in parts of Ottawa in more recent years (Figure 1).

**Table 1:** Incidence of Lyme disease and characteristics of 639 reported human Lyme disease case-patients in 3 public health units, eastern Ontario, Canada, 2010–2016*

Characteristic	*No. (%) cases*	*Cumulative incidence, cases/100,000 population†*	*Mean (SD) annual incidence, cases/100,000 population†*
Public health unit			
KFL	210 (33.0)	109.6	15.7 (13.3)
LGL	224 (35.1)	135.8	19.4 (10.4)
Ottawa	205 (32.1)	23.2	3.3 (2.8)
Total	639 (100.0)	51.5	7.4 (5.2)
Age group, y			
0–9	43 (6.7)	32.6	4.7 (4.5)
10–19	39 (6.1)	25.7	3.6 (2.2)
20–29	49 (7.7)	29.0	4.1 (3.3)
30–39	74 (11.6)	47.2	6.7 (4.2)
40–49	80 (12.5)	42.2	6.0 (5.0)
50–59	161 (25.2)	87.9	12.6 (8.5)
60–69	118 (18.5)	89.2	12.7 (9.9)
70–79	56 (8.8)	74.7	10.7 (8.8)
>80	19 (3.0)	38.1	5.4 (4.3)
Sex			
F	272 (42.6)	42.7	6.1 (4.5)
M	364 (57.0)	60.4	8.6 (6.1)
Data missing	3 (0.4)	–	–

*KFL, Kingston, Frontenac, Lennox, and Addington; LGL, Leeds, Grenville, and Lanark. †Population based on 2011 census.

**Table 2:** Incidence of Lyme disease and number of *Ixodes scapularis* ticks submitted through passive tick surveillance, by year, 3 public health units, eastern Ontario, Canada, 2010–2016*

Characteristic and public health unit	*2010*	*2011*	*2012*	*2013*	*2014*	*2015*	*2016*
Incidence rate, cases/100,000 population†							
KFL	2.1	8.4	4.2	8.4	20.4	36.0	30.3
LGL	5.5	9.1	16.4	24.2	21.2	37.0	22.4
Ottawa	0.2	0.7	1.8	4.1	2.4	7.6	6.5
Total	1.2	3.0	4.1	7.4	7.7	15.9	12.3
No. Ixodes scapularis tick submissions							
KFL	209	620	677	864	115	51	23
LGL	359	865	870	969	468	69	17
Ottawa	38	106	134	239	258	216	336
Total	606	1,591	1,681	2,072	841	336	386

*KFL, Kingston, Frontenac, Lennox, and Addington; LGL, Leeds, Grenville, and Lanark. †Population based on 2011 census.

Within our study area, the first human LD case was reported an average of 2.2 years after the first reported *I. scapularis* tick and 1.1 years after the first reported *B. burgdorferi*–infected tick. Time to first case was significantly associated with time to first reported *I. scapularis* tick (adjusted r^2^ = 0.56; p<0.001) and time to first *B. burgdorferi*–infected tick (adjusted r^2^ = 0.67; p<0.001) after adjusting for distance to the FSA with highest LD incidence in 2010. The associated lag between each phase of ≈1 year supports the hypothesis that invasion and establishment of tick populations is followed by colonization of *B. burgdorferi* (*12*), or it might reflect the arrival of infected ticks with subsequent increase in *B. burgdorferi* prevalence. However, drawing conclusions on the exact timing of tick and pathogen invasion is difficult because of the nature of passive surveillance data.

**Figure 2:**
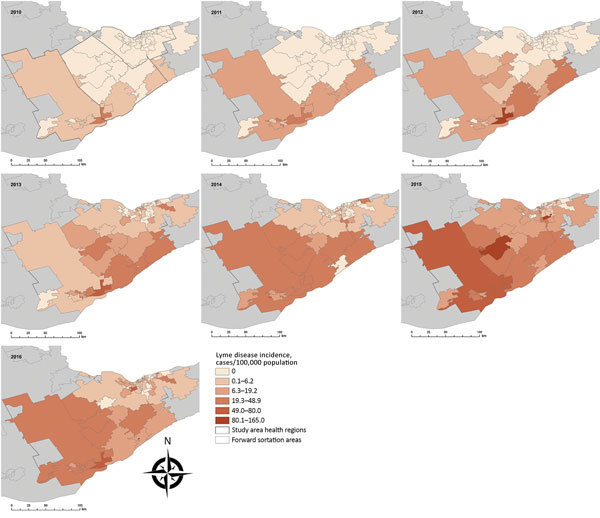
Spatiotemporal spread of human Lyme disease incidence, 3 public health units, eastern Ontario, Canada, 2010–2016. Annual Lyme disease incidence estimated from notifiable disease surveillance and population data based on forward sortation area of patient residence.

LD incidence was concentrated in southern FSAs in 2010 and 2011 but had spread in a northeasterly direction by 2013 (Figure 2). Overall, a northeast shift of 54 km occurred between mean centers during 2010–2016, with the greatest spread observed in 2011–2013 (Appendix). We detected a spatiotemporal cluster of high rates of *B. burgdorferi*–infected ticks in the Kingston-Gananoque region bordering the St. Lawrence River, which overlapped with 2 clusters of human LD cases (Appendix Figure 4). The overlapping clusters support the conclusion that increased tick encounter is a determinant of human LD risk. Residence in endemic areas (i.e., where infected ticks have been found) has been consistently recognized as a risk factor for LD infection (*13*,*14*).

## Conclusions

Although LD incidence in Ottawa had reached ≈7 cases/100,000 population by 2015–2016, the observed incidence rates in KFL and LGL during this period were 4-fold higher (≈30 cases/100,000 population). By comparison, these rates are still far below the ≈110 cases/100,000 population observed in the bordering St. Lawrence County of New York state (*15*). Given the ongoing emergence process, LD incidence will likely continue to increase in eastern Ontario as *I. scapularis* tick populations and *B. burgdorferi* continue to establish and fill in suitable habitats (*12*). This pattern highlights the importance of fine-scale studies to identify patterns and determinants of LD and other tickborne pathogens in different regions and populations.

Our study was limited by the availability of information on location of tick acquisition and patient exposure location. As such, we aggregated data at the FSA level on the basis of location of patient and tick submitter residence and excluded case-patients and tick submitters with reported travel outside their municipality of residence. Spatiotemporal analysis based on the location of exposure would help to more precisely determine the timing and rate of spread.

Altogether, our findings indicate that LD has emerged in eastern Ontario over a relatively short timescale after the invasion of *I. scapularis* ticks and *B. burgdorferi*. Tick surveillance data can serve to identify areas of risk for LD emergence.

Appendix Additional information on Lyme disease emergence after invasion of the blacklegged tick, *Ixodes scapularis*, Ontario, Canada, 2010–2016.
