# Supplementary material for: Lyme Disease Emergence after Invasion of the Blacklegged Tick, Ixodes scapularis, Ontario, Canada, 2010–2016
Source: Emerg Infect Dis. 2019 Feb;25(2):328–32. doi: 10.3201/eid2502.180771 (PMC6346472; doi:10.3201/eid2502.180771)
Supplement: Appendix — Additional information on Lyme disease emergence after invasion of the blacklegged tick, Ixodes scapularis, Ontario, Canada, 2010–2016. [file 18-0771-Techapp-s1.pdf]

Article DOI: https://doi.org/10.3201/eid2502.180771

Lyme Disease Emergence after Invasion of the Blacklegged Tick, *Ixodes scapularis*, Ontario, Canada, 2010–2016

Appendix

Supplementary Methods

Data Sources

Reportable Disease Surveillance Data

All public health units (PHUs) in Ontario are required to collect and report information on patients with reportable diseases to the Ministry of Health and Long-Term Care (MOHLTC). This information is entered into the Integrated Public Health Information System (iPHIS) database and used for local, provincial, and national surveillance. A map of the three PHUs included in the study is shown in Supplementary Figure 1. We defined cases as patients with confirmed or probable Lyme disease (LD) as defined by MOHLTC (*1*).

Tick Surveillance Data

Passive tick surveillance involves the voluntary submission of ticks by the public and by physicians and veterinarians from patients. Public Health Ontario (PHO) receives and identifies ticks that have been found on human hosts and submitted from public health units and/or directly from healthcare providers; these data are then collated with real-time polymerase chain reaction (PCR) test results from the National Microbiology Lab (*2*).

Statistical Analysis

We completed all statistical analyses using Stata 15; a two-sided significance level of 5% was used for statistical testing. We calculated LD incidence rates as the total annual number of cases in a given FSA divided by the FSA population. Similarly, we calculated annual *B. burgdorferi* prevalence as the total number of submitted *I. scapularis* ticks with positive real-

time PCR results for *B. burgdorferi* divided by the total number of ticks that were tested in a given FSA and year.

To examine the association between the invasion of *I. scapularis* and *B. burgdorferi* and the spread of human LD, we examined bivariable associations between FSA-level data on time to first case (in years) and the following independent variables: time to first reported *I. scapularis* tick, time to first reported *B. burgdorferi*-infected tick, distance to origin, and population. We constructed linear regression models with time to first case (in years) as the outcome and variables that were significant in bivariable analyses as predictors. The origin of LD was defined as the FSA with highest LD incidence in 2010, which corresponded to the town of Gananoque. We tested data for linearity and normality and examined model residuals to test goodness of fit.

Spatial Analysis

To visualize LD spread from 2010 to 2016, we plotted the annual FSA-level incidence of human LD and *B. burgdorferi* prevalence in ticks using ArcGIS v10.4. We excluded tick records with missing collection date, patient FSA, PCR test result, and records with reported history of travel. Similarly, we excluded human LD records with missing patient residence information or with a reported history of travel. We assessed the annual weighted mean center and directional distribution of human LD incidence using the spatial analyst extension in ArcGIS, following spatial projection of the data to preserve distance (*3*). The resulting weighted mean center location represents the geographic center of concentration of features (FSA centroids) weighted by annual rates, while the deviation ellipses indicate 1 SD of the geographic mean annual incidence, calculated as the incidence weighted average in space for each FSA, with incidence attributed to the FSA centroid.

Cluster Analysis

We applied Kulldorf's spatial scan statistics (*4*) to assess and compare spatiotemporal patterns in human LD incidence and *B. burgdorferi* prevalence in ticks at the FSA level (FSA centroids). We used a Poisson-based probability model to detect space-time clusters of human LD cases occurring over FSAs in the three PHUs, adjusting for FSA population size. We used a Bernoulli-based probability model to detect clusters of *B. burgdorferi* infected ticks, with infected and non-infected ticks representing cases and controls, respectively. Using SaTScanTM, we set the scan to detect clusters with high rates, with 10% or 50% maximum spatial cluster size

for human cases and ticks, respectively, and with a 1-year minimum temporal window size. Significance of clusters was based on 999 Monte Carlo replications using a p-value of <0.05.

Supplementary Results

Epidemiology of LD

From 2010 to 2016, there were a total of 762 reported cases of LD within the study area. Of these, we excluded 123 from the analysis based on a reported history of travel. Of the 639 cases that were retained, we observed a higher proportion among males (n = 364, 57.0%) than females (n = 272, 42.6%). Cases ranged in age from under 1 year to 93 years (mean = 47.1 years of age; SD±20.4) with a peak among adults aged 45–69 years. Despite similar total case numbers among the three health units, incidence rates were higher in KFL and LGL owing to their lower overall population (Supplementary Figure 2).

Among all FSAs in the study area, the first human LD case was reported an average of 2.0 years following the first reported *I. scapularis* tick, and 0.6 years following the first reported infected tick. However, two FSAs reported human cases before any *I. scapularis* ticks were detected through passive surveillance, and six FSAs reported human cases before detection of *B. burgdorferi* in submitted ticks. This discrepancy may be due to differences in sensitivity of tick surveillance or may reflect patient exposure outside of the FSA of residence, despite our restriction based on travel history. We conducted a sensitivity analysis excluding discrepant FSAs (i.e., FSAs with human cases reported before a signal from tick surveillance), and found 2.2 and 1.1 years respectively following the first reported tick or *B. burgdorferi*-infected tick.

In bivariable analyses, time to first human LD case (in years) was independently associated with time to first *I. scapularis* tick (r2 = 0.35, $p < 0.001$), time to first *B. burgdorferi*-infected tick (r2 = 0.61, $p < 0.001$) and distance to origin (r2 = 0.46, $p < 0.001$), but not population size ($p = 0.062$). Time to first tick and time to first *B. burgdorferi* infected tick were highly correlated (Spearman rank correlation 0.78), therefore separate models were fit for each predictor. In a multivariable regression, after adjusting for distance to origin, time to first case was significantly associated with time to first reported *I. scapularis* tick (adjusted r2 = 0.56, $p < 0.001$). Time to first *B. burgdorferi*-infected tick was also strongly associated with time to first human LD case after controlling for distance from origin (adjusted r2 = 0.67, $p < 0.001$).

Trends in Tick Surveillance

A total of 7760 *I. scapularis* ticks were recorded from 2010 to 2016 through passive surveillance activities in the study area. Of these, 233 of submitters reported recent travel (95 outside of Canada and 138 within Canada); we excluded these records from analysis. Of the 7527 ticks retained in analyses, almost half were submitted by individuals residing in LGL (n = 3617, 48.2%), followed by KFL (n = 2559, 34.1%) and OTT (n = 1395, 17.7%). The number of collected ticks increased annually until 2014, when KFL and LGL no longer accepted tick submissions from the public (although healthcare providers could still submit ticks to PHO). Ticks were received throughout the entire year, with bimodal peaks from April to June and October to November reflecting the submission of mainly adult ticks, which are easier to see and more likely to be submitted than nymphal ticks.

Of the 5753 ticks with real-time PCR results for *B. burgdorferi*, 1209 (21.0%) tested positive; 1774 records had no test results reflecting ticks that were not forwarded for testing due to damage or poor specimen condition. The overall (cumulative) *B. burgdorferi* prevalence in ticks was similar among health units, with 21.6%, 21.0%, and 19.5% prevalence in KFL, LGL, and OTT, respectively. There was a significant association between infection status (positive/negative) and year of tick submission ($p < 0.001$).

Spatial and Cluster Analysis

We excluded 45 cases due to missing patient residence information and retained 594 human cases in spatial analyses. Deviational ellipses depict the spatiotemporal trend in LD incidence, which was concentrated in southern FSAs in 2010 and 2011 but had spread in a northeasterly direction by 2013 (Supplementary Figure 3). The directional trend was consistent from 2013 to 2016 reflecting the occurrence of reported LD cases in OTT from 2013 onward. Distances between mean centers, reflecting the change in LD distribution from year to year, increased from 2010 to 2013: 4.3 km (2010 to 2011), 20.9 km (2011 to 2012) and 35.8 km (2012 to 2013) (mean 20.3 km/year) and stabilized thereafter. Overall, a northeast shift of 54 km was observed between mean centers from 2010 to 2016.

We excluded 706 tick records with missing submitter residence information and retained 5047 records in spatial analysis; 1068 (21.1%) were positive for *B. burgdorferi*.

Space-time Poisson analysis revealed six clusters of high human LD incidence, two of which were significant ($p < 0.001$). The first significant cluster of human infection was 66 km in radius, was composed of 3 FSAs (including one geographically large FSA in KFL) covering a population of 93,811 and occurred over the period from 2014 to 2016. The incidence of LD within the cluster was 42.6 per 100,000, with a relative risk of 7.9. The second significant cluster of human infection centered on the Kingston-Gananoque region was 32 km in radius, was composed of 5 FSAs covering a population of 126,827, and also occurred over the period from 2014 to 2016. The incidence of LD within the cluster was 26.8 per 100,000, with a relative risk of 4.7. A similar pattern was observed in the space-time Bernouilli model, which revealed seven clusters of high rates of *B. burgdorferi* infected ticks, with one significant cluster ($p < 0.0001$). The significant cluster was 77 km in radius spanning 9 FSAs and overlapping spatially with the clusters of human LD cases. The infected tick cluster contained 28.5% of all infected ticks with RR = 1.6, and was observed over the period 2012 to 2014.

References

1. Ministry of Health and Long-Term Care. Infectious disease protocol appendix B: provincial case definitions for reportable diseases. Toronto: Ministry of Health and Long-Term Care; 2017.

2. Nelder MP, Russell C, Lindsay LR, Dhar B, Patel SN, Johnson S, et al. Population-based passive tick surveillance and detection of expanding foci of blacklegged ticks *Ixodes scapularis* and the Lyme disease agent *Borrelia burgdorferi* in Ontario, Canada. PLoS One. 2014;9:e105358. PubMed http://dx.doi.org/10.1371/journal.pone.0105358

3. Mitchell A. The ESRI guide to GIS analysis. Vol. 2. Redlands (California): ESRI; 2005.

4. Costa MA. Kulldorf M. Applications of spatial scan statistics: a review. In: Scan statistics: methods and applications. Boston: Birkhäuser]; 2009. p. 129–52.

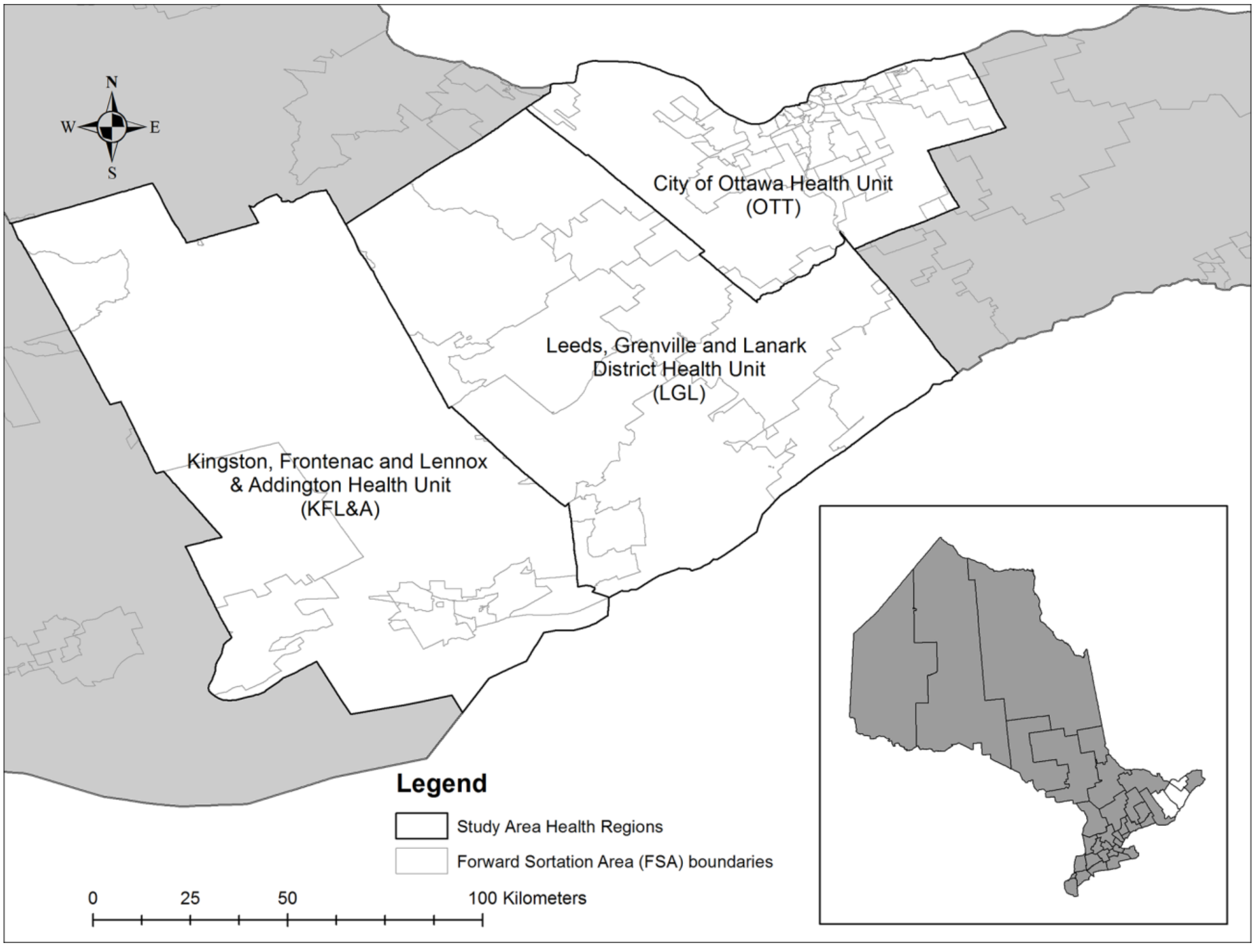

Appendix Figure 1. Map of study area showing three public health units in eastern Ontario, Canada. Inset map shows the province of Ontario with the study area location in white.

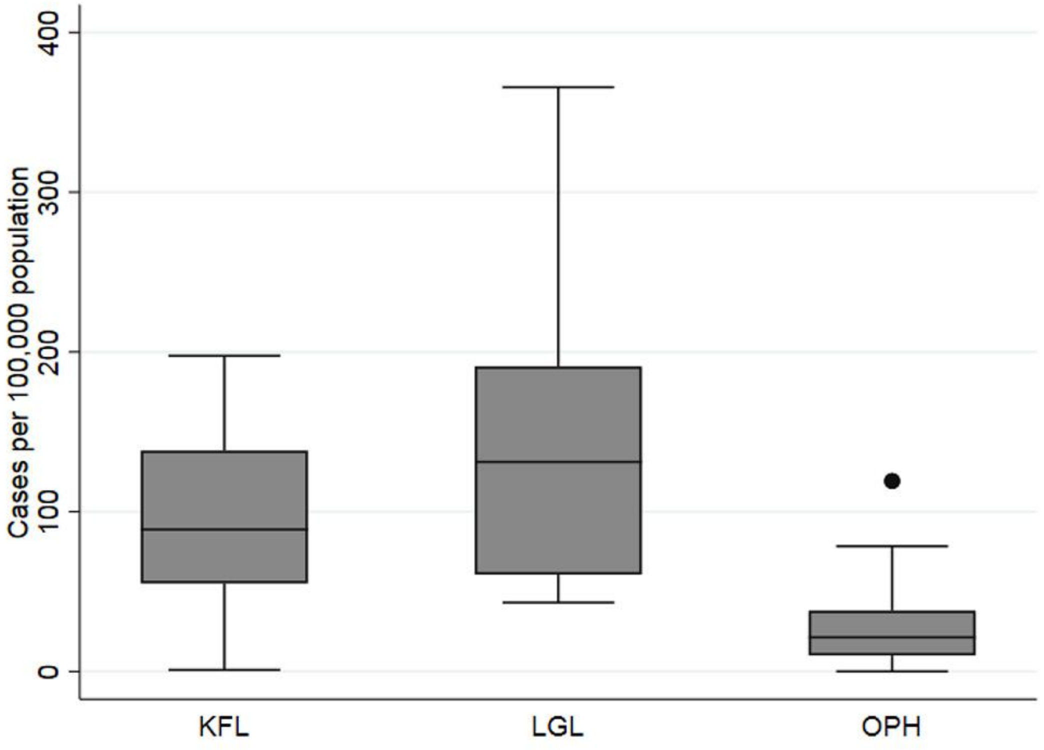

Appendix Figure 2. Boxplot of cumulative Lyme disease incidence, 2010–2016, in three eastern Ontario public health units. Error bars show 95% confidence intervals and point indicates outlier data, based on data from forward sortation areas in each health unit; Kingston, Frontenac, Lennox & Addington (KFL), Leeds, Grenville, Lanark (LGL), Ottawa (OTT).

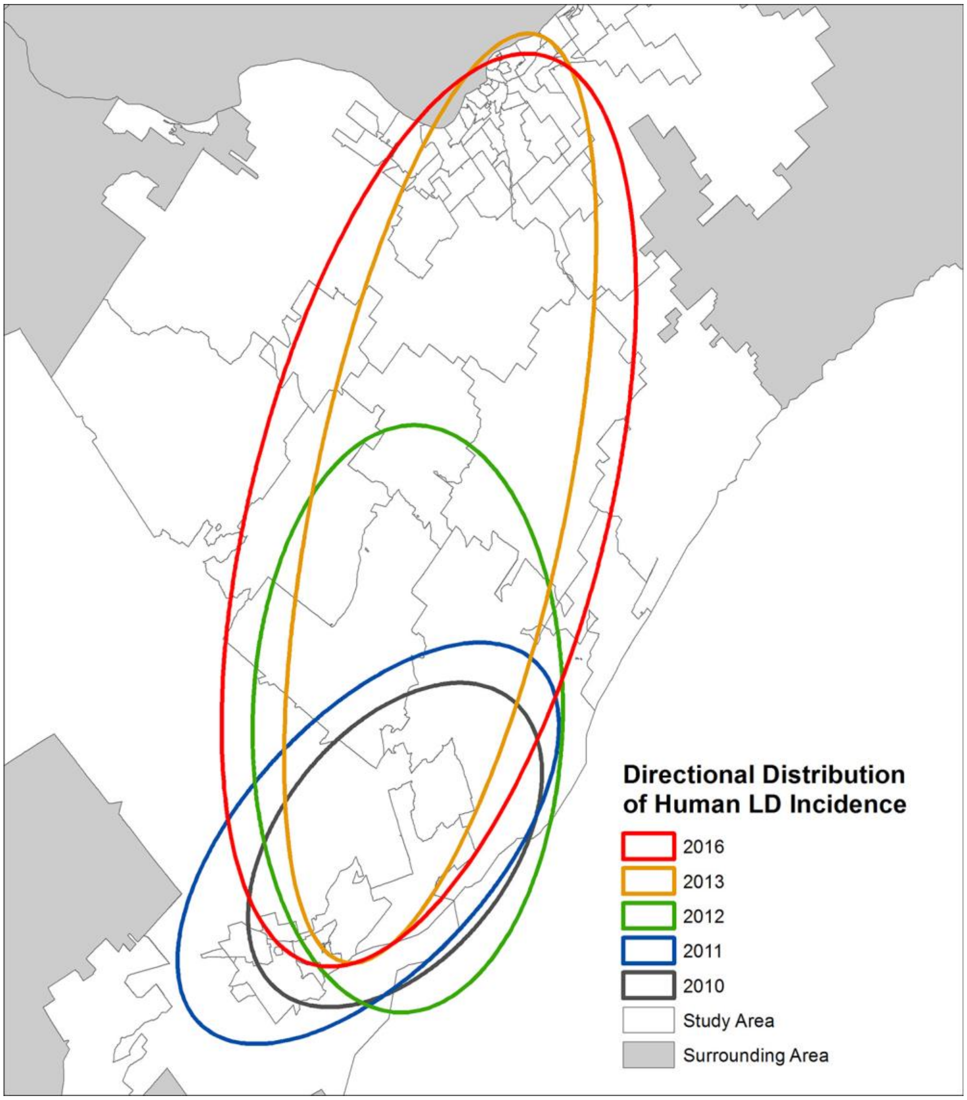

Appendix Figure 3. Deviational ellipses indicating the directional distribution of human Lyme disease incidence in three eastern Ontario public health units, 2010–2016.

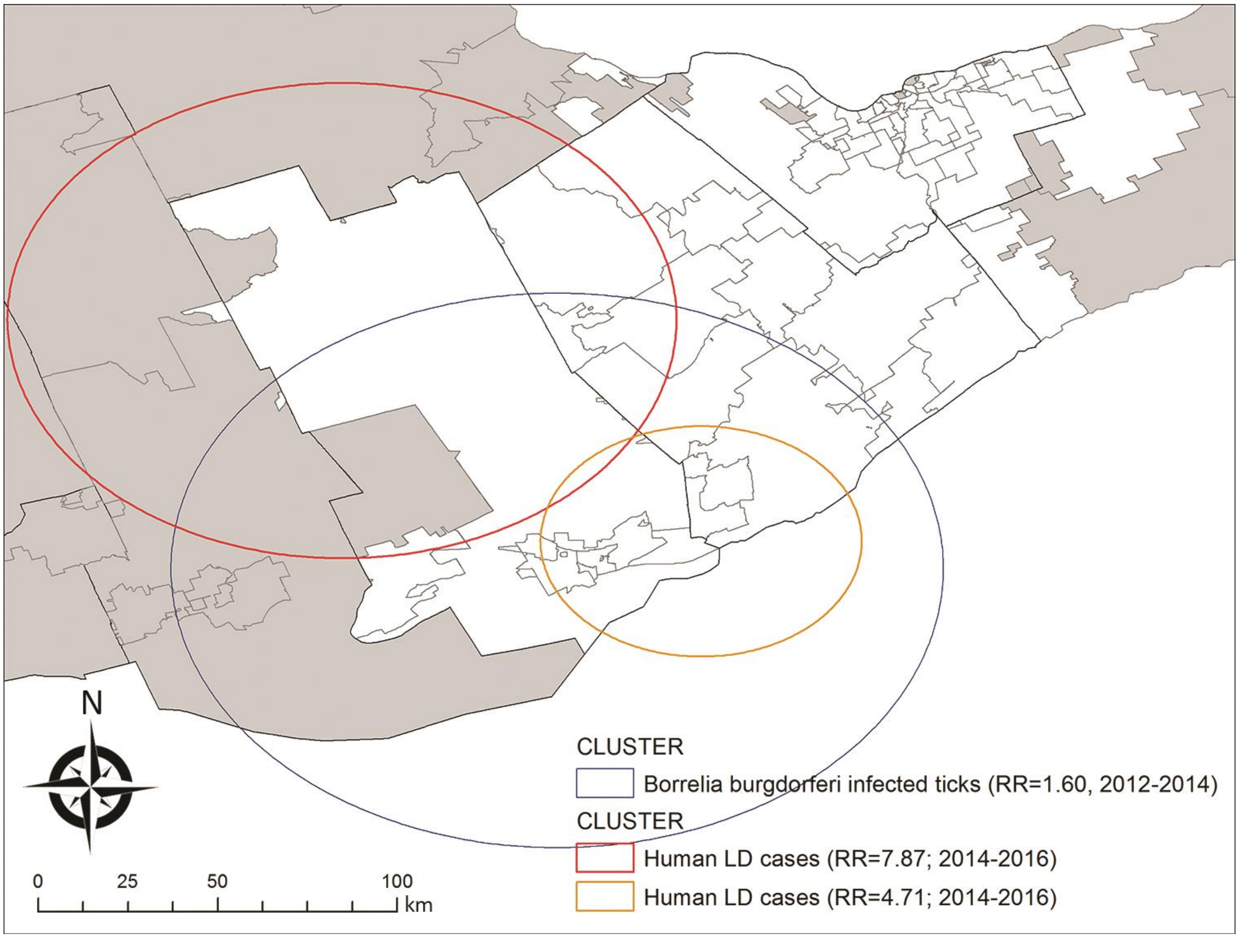

Appendix Figure 4. Spatiotemporal clusters of high rates of *Borrelia burgdorferi*–infected ticks (dark blue outline) and human Lyme disease cases (yellow and red outlines) in three eastern Ontario public health units, 2010–2016. The relative risk (RR) and duration of observed clusters (start and end year) are indicated in the figure legend.
